# Pro-angiogenic and osteogenic composite scaffolds of fibrin, alginate and calcium phosphate for bone tissue engineering

**DOI:** 10.1177/20417314211005610

**Published:** 2021-04-06

**Authors:** Nupur Kohli, Vaibhav Sharma, Alodia Orera, Prasad Sawadkar, Nazanin Owji, Oliver G Frost, Russell J Bailey, Martyn Snow, Jonathan C Knowles, Gordon W Blunn, Elena García-Gareta

**Affiliations:** 1 Regenerative Biomaterials Group, The RAFT Institute & The Griffin Institute, Northwick Park & Saint Mark’s Hospital, London, UK; 2 Department of Mechanical Engineering, Imperial College London, London, UK; 3 Instituto de Nanociencia y Materiales de Aragón (INMA), CSIC-Universidad de Zaragoza, Zaragoza, Spain; 4 Division of Biomaterials and Tissue Engineering, UCL Eastman Dental Institute, University College London, London, UK; 5 The NanoVision Centre, School of Engineering and Materials Science, Queen Mary University of London, London, UK; 6 Royal Orthopaedic Hospital NHS Foundation Trust, Birmingham, UK; 7 Department of Nanobiomedical Science & BK21 Plus NBM Global Research Centre for Regenerative Medicine, Dankook University, Cheonan, Republic of Korea; 8 The Discoveries Centre for Regenerative and Precision Medicine, UCL Campus, London, UK; 9 UCL Eastman-Korea Dental Medicine Innovation Centre, Dankook University, Cheonan, Republic of Korea; 10 School of Pharmacy and Biomedical Sciences, University of Portsmouth, Portsmouth, UK

**Keywords:** Bone tissue engineering, angiogenic, osteogenic, fibrin, calcium phosphate

## Abstract

Due to the limitations of bone autografts, we aimed to develop new composite biomaterials with pro-angiogenic and osteogenic properties to be used as scaffolds in bone tissue engineering applications. We used a porous, cross-linked and slowly biodegradable fibrin/alginate scaffold originally developed in our laboratory for wound healing, throughout which deposits of calcium phosphate (CaP) were evenly incorporated using an established biomimetic method. Material characterisation revealed the porous nature and confirmed the deposition of CaP precursor phases throughout the scaffolds. MC3T3-E1 cells adhered to the scaffolds, proliferated, migrated and differentiated down the osteogenic pathway during the culture period. Chick chorioallantoic membrane (CAM) assay results showed that the scaffolds were pro-angiogenic and biocompatible. The work presented here gave useful insights into the potential of these pro-angiogenic and osteogenic scaffolds for bone tissue engineering and merits further research in a pre-clinical model prior to its clinical translation.

## Introduction

The current treatment options for restoring bone defects involve the use of autologous bone grafts, allogeneic bone grafts and alloplastic materials. As it stands, autograft bone substitute is benchmarked as the gold standard treatment for offering osteoinductive and osteoconductive properties. However, this is associated with complications such as donor site morbidity, limited bone supply and increased risk of infection. As a result, many attempts have been made to fabricate bone implants that can offer suitable biological and mechanical properties. Similarly, the emergence of bone tissue engineering has led to the introduction of novel strategies that can serve as effective bone graft substitutes. Nevertheless, successful translation of such techniques, still remains a significant challenge.^1^

The naturally occurring biodegradable polymer fibrin is the first bio-scaffold built by our body following tissue injury. Formation of a fibrin scaffold initiates haemostasis and provides a temporal matrix that supports cellular activity, including deposition of a new extracellular matrix (ECM). For tissue engineering applications, fibrin possesses excellent biocompatibility, pro-angiogenic and bioactive properties. However, its bio-degradable and mechanical properties are poor, thus limiting its use.^2,3^ Nevertheless, the pro-angiogenesis of the material makes it attractive for regeneration of highly vascularised tissues such as bone.^4–9^ In bone repair and regeneration, the main strategy is to combine fibrin with other polymers to create composites that in some cases include a ceramic component.^4,5^ Alginate is an anionic polysaccharide that conversely does not have the excellent biological properties of fibrin. Nevertheless, when in contact with divalent ions like Ca^2+^, alginate is cross linkable, allowing the polysaccharide to maintain its form.^10^ When both fibrin and alginate are combined to form a composite scaffold the limitations of both biomaterials are overcome. By cross-linking the composite material, its biodegradation is slowed and the mechanical properties are enhanced. Our laboratory has developed a porous, cross-linked fibrin/alginate scaffold for the treatment of acute full thickness skin wounds and is currently in clinical trials.^11–14^ The scaffold has exhibited promising advantages in terms of retarding degradation and improving cell ingress, thus enhancing tissue regeneration and establishment of a blood supply.^11,12^ Since angiogenesis is key for successful bone regeneration,^2,3^ we hypothesized that our pro-angiogenic fibrin/alginate scaffold could be used for bone tissue engineering applications by adding an osteogenic component to the scaffold, such as a calcium-phosphate (CaP).

The biomineral present in bone is a CaP phase that has been defined as ‘a poorly crystalline, highly substituted apatite consisting of very small crystals’.^15,16^ Implants made of or incorporating CaP, or coated with it, show improved interaction with the surrounding bone tissue. Biomaterials incorporating CaP are often regarded as osteoconductive and sometimes they can be osteoinductive as well.^1^ However, a current limitation of CaP materials is their composition. The nanometer-sized platelet or needle crystals in bone incorporate several ionic substitutions, for example, Na^+^, Sr^2+^ and Mg^2+^ in Ca^2+^ sites or CO_3_^2–^ in OH^–^ (A-substitution) and PO_4_^3–^ sites (B-substitution). Currently, CaP phases used for bone repair that is, hydroxyapatite [Ca_10_(PO_4_)_6_(OH)_2_ with Ca/P = 1.67] or β-tricalcium phosphate [β-Ca_3_(PO_4_)_2_ with Ca/P = 1.50] do not possess the chemical versatility of bone mineral. Consequently, efforts are being spent on producing CaP materials consisting of precursor phases that is, amorphous calcium phosphate [Ca_3_(PO4)_2_·*z*H_2_O where *z* = 3–4.5 in basic conditions and M_3_(Ca_3_(HPO_4_)_4.5_·*z*H_2_O) where *z* is unknown and M is typically a monovalent cation (Na^+^, K^+^, NH_4_^+^) in acidic conditions; Ca/P = 0.67–1.50] or octacalcium phosphate [Ca_8_H_2_(PO_4_)_6_·5H_2_O with Ca/P = 1.33]. These precursor CaP phases can be rapidly converted to apatite after implantation.^1,15^ However, production of these precursor phases in granular form has limited usability. Therefore, combination of them with a bioactive polymeric fibrous matrix would increase their clinical uses, shown by Chahal et al.^17^ and Kawai et al.^18^ Such precursor phases could be deposited on a bioactive matrix like our fibrin/alginate scaffold by biomimetic deposition following immersion in simulated body fluids.^19,20^

The aim of this study was to develop new composite biomaterials with pro-angiogenic and osteogenic properties to be used as scaffolds in bone tissue engineering applications. For this purpose, a porous, cross-linked and slowly biodegradable fibrin/alginate scaffold originally developed in our laboratory for wound healing applications was used, throughout which deposits of CaP precursor phases were evenly incorporated using an established biomimetic method.

## Materials and methods

### Preparation of composite scaffolds

Composites were prepared by immersing the fibrin/alginate (FA) scaffolds in 5× concentrated simulated body fluid (SBF) solutions resulting in two prototypes.^19,20^ The FA scaffolds were previously manufactured in our laboratory using custom made methods.^11–14^ Briefly, reagents needed for manufacturing the FA matrix^21^ were whisked into a white foam that was casted on a mould where the foam was allowed to clot for 1 h at 37°C before chemical crosslinking with 0.2% vol/vol glutaraldehyde (Sigma-Aldrich, UK) in 80% ethanol/20% MES [2-(N-morpholino ethanesulfonic acid (69889, Sigma UK), 0.1 M, pH = 7.4] buffer for 4 h at room temperature. The scaffolds were then washed with 0.1% wt/vol sodium borohydride (452882, Sigma-Aldrich, UK) in diH_2_O and diH_2_O at room temperature, and finally lyophilised for 36 h at −40°C (Virtis Genesis Freeze Dryer, Biopharma, UK).

Fibrin/alginate-CaP1 (FACaP1) was prepared by immersing fibrin/alginate scaffolds in SBF-1 solution comprising, 0.695 g of CaCl_2_; 0.760 g of MgCl_2_.6H_2_O; 0.880 g of NaHCO_3_ and 0.570 g of K_2_HPO_4_.3H_2_O added to 0.4 L of distilled water (dH_2_O). Following this, the pH of the solution was adjusted to 6.0 using 1M HCl, after which, 0.560 g of KCl and 20.135 g of NaCl were added to the solution. Finally, the pH of the solution was adjusted to 6.5 using 1 M NaOH. 1 × 1 cm pieces of FA scaffolds were immersed in SBF-1 solution for 24 h with constant stirring at 150 rpm on an orbital shaker at 37°C. After the immersion time, scaffolds were removed from the SBF-1 solution, washed with dH_2_O in an ultrasonic water cleaner for 60 s, frozen at −80°C and lyophilised.

To prepare the fibrin/alginate-CaP2 (FACaP2) scaffolds, the dried FACaP1 scaffolds were immersed in SBF-2 solution, comprising 20.135 g of NaCl, 0.695g of CaCl_2_ and 0.570 g of K_2_HPO_4_.3H_2_O added to 0.4 L of dH_2_O. The pH of this solution was adjusted to 6.0 with 1 M HCl. The scaffolds were immersed in SBF-2 solution for 48 h with constant stirring at 60 rpm on an orbital shaker at 37°C. After the immersion time, scaffolds were washed, frozen and lyophilised.

All solutions were filtered with a 0.22 µm PES membrane before use. FA scaffolds immersed in dH_2_O were used as controls. All scaffolds were weighed pre and post-immersion to examine a change in the net weight as a result of the coating procedure.

### Material characterisation of composite scaffolds

#### Scanning electron microscopy (SEM) and energy dispersive X-ray spectroscopy (EDX)

Lyophilised scaffolds were carbon coated before observation under SEM and EDX analysis. SEM microphotographs were taken at 100× and 10,000×, and obtained at 10 kV using the Inspect F, FEI Company, The Netherlands. Morphology of CaP crystal deposits was studied from the SEM images. Elemental analysis of the scaffolds and Ca/P ratio were studied from the EDX spectra that were obtained at 10 kV using the X-Act, Oxford Instruments, UK.

#### Fourier transform infrared spectroscopy (FTIR) and x-ray diffraction (XRD)

Analysis of functional groups in the CaP deposits was carried out by FTIR. Spectra were obtained by placing the scaffolds in contact with Attenuated Total Reflectance accessory (Golden Gate ATR, Specac, UK). Spectrum software v 5.0.1 (Perkin-Elmer, UK) identified the peak intensities of each chemical group (the wavenumber was fixed between 500 and 4000 cm^−1^ with a resolution of 4 cm^−1^).

Phase composition and crystallinity of the CaP deposits were studied by XRD using a RIGAKU D/max 2500 Diffractometer operated at 40 kV and 80 mA with graphite-filtered Cu Kα radiation. Data was collected from 2θ = 5° to 80° with a step size of 0.03°

#### Rheology

To examine the viscoelastic properties of the scaffolds, we used a Kinexus Rheometer (Malvern Instruments, UK) in its oscillatory mode. Hydrated 2 × 2 cm pieces of samples were placed between two 20 mm diameter parallel plates. There was a 0.3 mm gap between the plates. One sample was measured at a time. An integrated temperature controller was used to maintain the temperature of the sample stage at 20°C. The ‘amplitude sweep’ and the ‘frequency sweep’ measurement was carried out on each sample. The ‘amplitude sweep’ was performed by applying controlled stresses that were linearly increased from 0.05% to 5%. Strains corresponding to the stresses were recorded. The oscillatory frequency was maintained at 1 Hz. The maximum strain within the linear viscoelastic region (LVER) was chosen from the ‘amplitude sweep’. The shear or storage modulus G′ was calculated for all the samples.

#### Mercury intrusion porosimetry (MIP)

Samples were outsourced to MCA services (Royston, UK) for this analysis. Briefly, samples were weighed into a Micromeritics AutoPore V 9620 penetrometer. Blank correction was applied using a reference analysis of this penetrometer under the same analytical conditions. The assembled penetrometer was weighed with and without mercury. Sample evacuation was conducted to 50 mmHg. Intrusion data were collected in the approximate applied pressure range 0.3–60,000 psia with equilibration by time (5 s). Maximum mercury intrusion limits were set to 0.01 mL/g or lower to ensure collection of sufficient data points in regions of mercury intrusion.

#### Histology and Von Kossa staining

To assess deposition of CaP throughout the depth of the FA scaffolds, histology followed by Von Kossa staining was carried out. Samples were processed, embedded in paraffin wax and sectioned into 4 µm thick sections. The sections were adhered to glass slides and placed in a hotbox at 60°C for 25 min. Once the sections were dry, Von Kossa staining was conducted. The principle of the Von Kossa staining is a precipitation reaction in which silver ions react with phosphate under acidic conditions. Then, photochemical degradation of silver phosphate to silver occurs under light illumination. Slides were covered with 1.5% silver nitrate solution and exposed to bright light for 1 h (under a lamp), after which they were washed with dH_2_O. Then, slides were covered with 2.5% sodium thiosulphate for 5 min and dipped in running water before immersion in Eosin counter stain for 5 min. Slides were dipped in 70% IMS, then 90% IMS and immersed in 100% IMS for 1 min. Finally, they were immersed in Xylene for 2 min, dipped twice in Xylene and then a coverslip was placed using DPX mounting media, for observation under light microscopy. CaP deposits were stained black/dark brown while FA fibres were stained pink/red.

#### In vitro biodegradability

Scaffolds were cut into 5 × 5 mm square pieces prior to treatment with 0.5% trypsin at pH 7.2 at 37°C for up to 72 h. Scaffolds were immersed in PBS alone as controls. Demineralised bone matrix (DBM) 5 × 5 mm square pieces were also used as a control to examine how quickly or slowly FACaP scaffolds degrade in comparison to this clinically used material. Samples were imaged macroscopically using canon camera and microscopically using a stereomicroscope at 0, 18, 24, 42, 48 and 72 h.

### In vitro cell work

#### In vitro response of inflammatory cells

As previously suggested by our group,^22^ we seeded macrophages on scaffolds to examine a difference in the pro-inflammatory nitric oxide (NO) production. RAW264.7 cell line was cultured as per the manufacturer’s guidelines (Sigma, UK). At passage 2, 1 × 10^5^ cells were directly seeded onto the scaffolds, previously cut into 5mm x 5mm pieces. The scaffolds were sterilised using 70% ethanol, followed by three washes in PBS, prior to cell seeding. Two h after cell seeding, 1 ml of DMEM media with or without 1 µg/ml of lipopolysaccharide (LPS) was added to the wells. After 24 h of incubation at 37°C, media was harvested for the analysis of NO production using Griess reagent system (Promega, UK). Briefly, 50 μl of media from the wells was added to a 96-well plate, followed by 50 μl sulphanilamide and 50 μl N-1-napthylethylenediamine dihydrochloride (NED). Absorbance at 520 nm was measured by microplate reader and nitrite concentrations were calculated using a standard nitrite curve.

#### Cell seeding and culture of osteoprogenitor cells

MC3T3-E1 subclone mouse pre-osteoblasts (osteoprogenitor cells) were used in our study. Scaffolds were cut into 5 × 5 mm pieces and sterilised with 70% IMS, washed three times with PBS and placed in flat bottomed-24 well ultra-low attachment plates. The scaffolds were seeded with 1 × 10^5^ MC3T3-E1 cells in 20 µl medium. After seeding, the plates were incubated for 3 h at 37°C with 5% CO_2_ to allow cells to attach to the scaffolds. Then, 1 ml of αMEM (Minimum Essential Medium Eagle Alpha Modification with 1% antibiotics and 10% fetal calf serum) with (+OM) or without (−OM) osteogenic supplements (50 µg/ml ascorbic acid, 10 mM β-glycerophosphate and 100 nM dexamethasone) was added per well and cultured over a 28-day period at 37°C with 5% CO_2_. Cell viability, proliferation, infiltration and osteogenic differentiation under both +OM and −OM conditions were assessed as described in the next sections.

#### Cell viability and proliferation of osteoprogenitor cells

Seeded scaffolds were assessed for cell incorporation and viability using Live/Dead cell staining according to the manufacturer’s guidelines (Sigma), wherein live cells fluoresce green and dead cells fluoresce red. Briefly, scaffolds were washed in PBS prior to staining with the live/dead staining solution and then the staining procedure was performed in the dark for 30 min at 37°C and 5% CO_2_. Live and dead cells were visualized by fluorescence imaging and confocal microscopy.

Seeded scaffolds were transferred to fresh 24 well plates and 1 ml of alamarBlue® working solution (diluted 10× from stock solution with phenol free Dubelcco’s Modified Eagles Medium supplemented with 10% FCS and 1% antibiotics) was added per well. Samples were incubated at 37°C with 5% CO_2_ for 3 h, after which each wells’ content was transferred to a cuvette and absorbance measured at 570 nm in a UV/vis spectrophotometer.

#### Infiltration and migration of osteoprogenitor cells

Seeded scaffolds were fixed in 10% formalin, processed and cut into 4 µm sections that were deparaffinized, rehydrated and washed in distilled water before applying Fluoroshield™ with DAPI mounting media (F6057, Sigma, Gillingham, UK) that was left to set for 5 min at room temperature. The sections were then cover slipped and the edges sealed with nail varnish before imaging with a confocal scanning laser microscope (Leica DMIRE2, Leica, Wetzlar, Germany). Sections were tile-scanned for the entire XY plane of the scaffold and then merged using 5% overlap automated function of the confocal microscope.

#### Osteogenic differentiation of osteoprogenitor cells

Osteopontin is a non-collagenous bone ECM protein that is commonly used as an early marker of osteogenic differentiation and in this study was assessed by osteopontin immunostaining of paraffin-embedded sections. Briefly, antigen retrieval was performed by heat mediated antigen retrieval using sodium citrate buffer at pH 6. After antigen retrieval the sections were blocked using 5% bovine serum albumin at 35°C for 1 hour and stained with primary anti-osteopontin antibody (1:50, abcam ab8448) at 4°C overnight. Following this, the sections were stained using the Alexa fluor 546 secondary antibody for 2 h at room temperature. The sections were then washed and mounted with DAPI based mounting media, cover slipped and visualised using Leica DMIRE2 confocal microscope (Leica, Wetzlar, Germany). Furthermore, mineralisation was assessed with Von Kossa staining of paraffin-embedded sections as previously described.

### Ex ovo chorioallantoic membrane (CAM) assay

Pro-angiogenic potential of the scaffolds was assessed using an ex ovo CAM assay previously reported by our group.^23^ Briefly, scaffolds were cut into 5 × 5mm square pieces, sterilised with 70% IMS and washed three times with PBS. Filter paper discs soaked in either PBS (negative) or 10 ng/ml of vascular endothelial growth factor (VEGF) solution (positive) were used as controls. Fertile chicken eggs were incubated at 37.5°C and 35%–45% humidity in an egg incubator. At day 3 post-incubation, the embryos were transferred to a shell-less culture system with 75%–80% humidity and 37.5°C incubation temperature. At embryonic day (ED) 9, scaffolds were applied onto the developing CAMs and incubated. On ED12, scaffolds were excised following cryopreservation and 4% paraformaldehyde fixation. Angiogenesis was examined in all the scaffolds macroscopically by taking photos using a stereomicroscope. ImageJ software was used to analyse the macroscopic photos and calculate the vascular density and the number of bifurcation points for each scaffold. Scaffolds were sectioned to assess blood vessel infiltration using H&E staining.

### Statistical analysis

GraphPad Prism 7 was used to analyse data. A minimum of *n* = 3 scaffolds were analysed per sample tested, unless otherwise stated and the data has been presented as mean ± standard error (SEM) of the mean. An Unpaired *t*-test with Welch’s correction was used to compare the differences in degradation of treated versus control scaffolds, and to compare NO production between activated and non-activated raw 264.7 cells. A non-parametric Dunn’s multiple comparison test was used to compare the differences in metabolic activity and percentage vascular area for each biomaterial tested. A one-way ANOVA was used to compare differences in the number of bifurcation points. A *p* value of <0.05 was considered significant.

## Results

### Material characterisation of composite scaffolds

#### Macroscopic appearance, SEM and EDX

**Figure 1.**
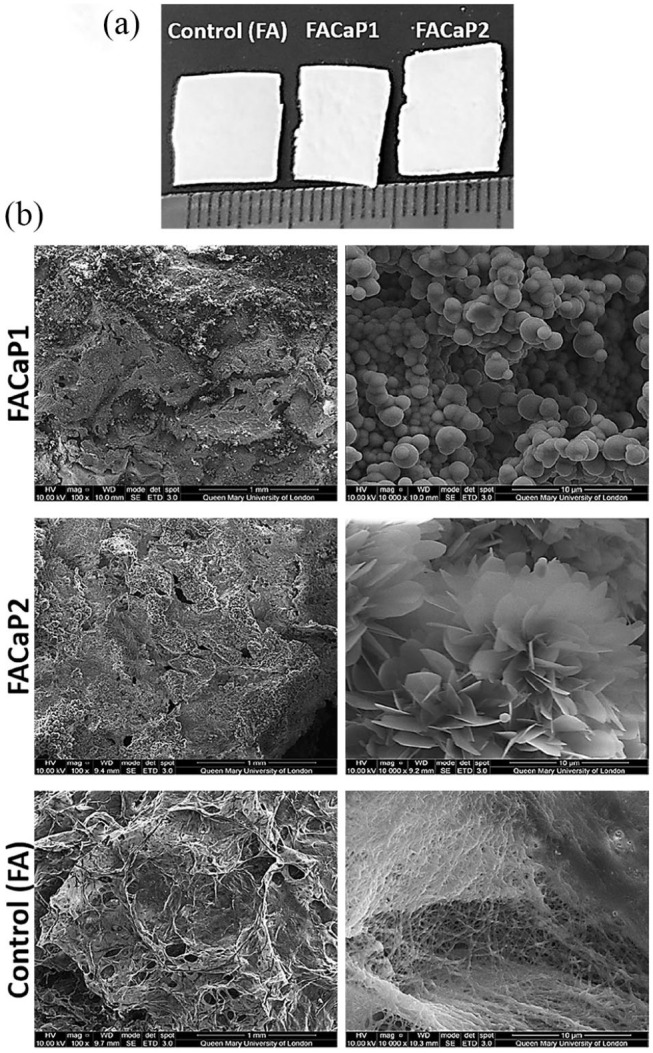
(a) Macroscopic appearance of scaffolds cut to approximately 1 × 1 cm pieces. Scale is in mm. (b) SEM representative images of control (FA) and FACaP scaffolds. Low magnification (100×) images on the left show the porous structure of the scaffolds. High magnification (10,000×) images on the right show the morphology of the mineral coatings on both FACaP1 and FACaP2 scaffolds. Control scaffolds showed no mineral deposition.

Macroscopically, scaffolds appear as white-coloured sheet meshes (Figure 1(a)), undistinguishable from each other. However, when handling them, the FACaP scaffolds feel stiffer and tougher than FA ones. Scaffolds can be manufactured to varying sizes.

SEM showed that FACaP1 and FACaP2 scaffolds contained mineral deposits throughout their surfaces. The mineral deposits in FACaP1 exhibited a globular morphology, whereas in FACaP2, a plate-like morphology was seen (Figure 1(b)). SEM images also showed that FACaP scaffolds remained porous after mineral deposition. The control FA scaffolds did not show mineral deposition and the fibres of the material were distinctively visible (Figure 1(b)).

**Figure 2.**
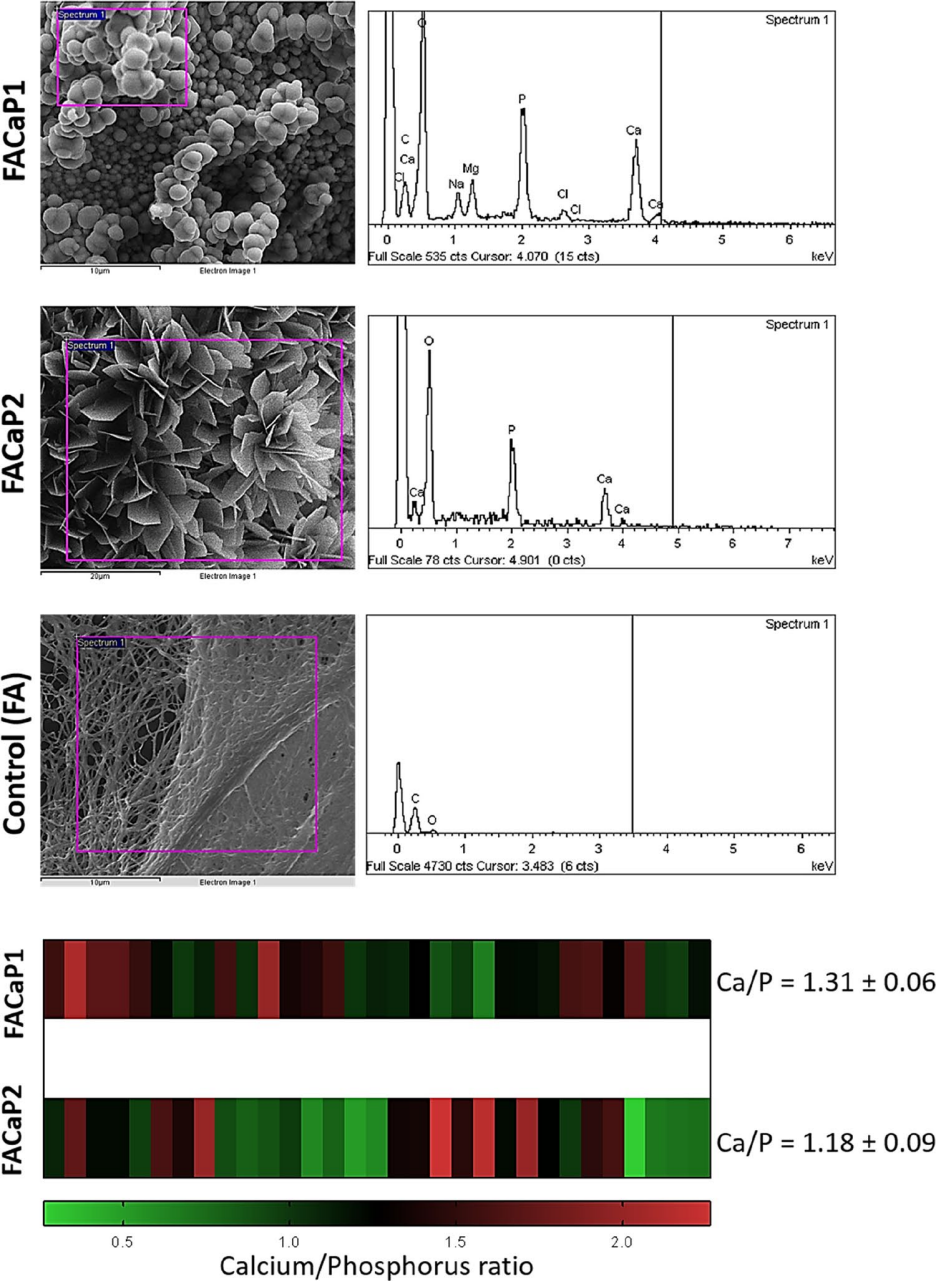
Representative EDX spectra and Ca/P ratio of the scaffolds, showing peaks for Ca and P in both FACaP1 and FACaP2 along with the presence of Mg in FACaP1. The control FA scaffolds did not show Ca and P peaks. The heatmap at the bottom displays the range of Ca/P ratio observed for multiple replicates of FACaP1 and FACaP2.

The EDX spectra revealed that the main elements in the mineral coatings of both FACaP1 and FACaP2 were Ca and P. In FACaP1 scaffolds, magnesium (Mg) was also present along with traces of sodium (Na) and chlorine (Cl) (Figure 2). The Ca/P ratio calculated for FACaP1 was 1.31 ± 0.06 and for FACaP2 was 1.18 ± 0.09 (mean ± SEM of multiple replicates). The control FA scaffolds did not show presence of Ca or P elements (Figure 2).

#### FTIR and XRD

**Figure 3.**
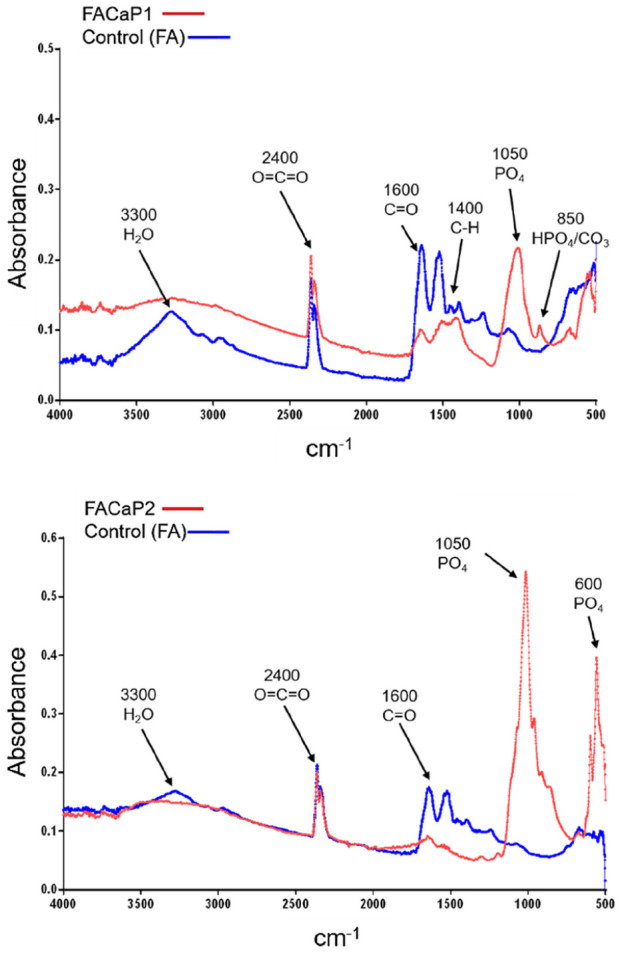
Representative FTIR spectra of scaffolds showing differences between control and FACaP scaffolds with PO_4_ and CO_3_ peaks seen in FACaP scaffolds only. A higher intensity peak for PO_4_ was seen in FACaP 2 compared to FACaP 1.

FTIR spectra (Figure 3) showed the difference in FACaP versus control scaffolds. Phosphate group (PO_4_) burst peaks were seen for FACaP1 samples at approximately 1050 cm^−1^ followed by a small shoulder peak at approximately 850 cm^−1^ which could be due to a hydrogen phosphate (HPO_4_) group or a carbonate (CO_3_) group or both. The small peak seen at approximately 1450 cm^−1^ for FACaP1 could be due to a CO_3_ group. A peak at approximately 600 cm^−1^, especially visible for FACaP2 samples, which could be due to a PO_4_ group. Moreover, spectra showed that PO_4_ peaks grow in intensity in FACaP2 samples compared to FACaP1 samples, suggesting deposition of a higher amount of a phosphate mineral phase in FACaP2. FTIR results suggested the presence of PO_4_ in FACaP scaffolds, and possibly both HPO_4_ and CO_3_ groups in FACaP1.

**Figure 4.**
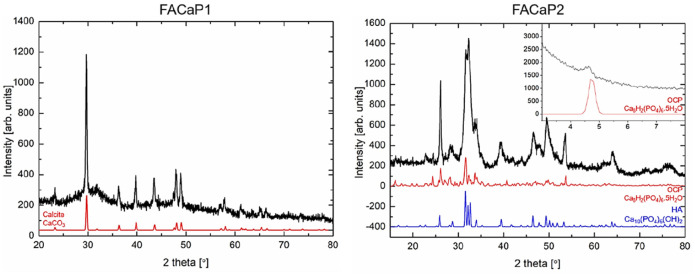
Representative XRD diffractograms of scaffolds. FACaP1 showed peaks for mainly calcite, with its reference pattern shown in red. FACaP2 scaffolds showed peaks mainly for OCP (reference pattern in red) and HA (reference pattern in blue) with an inset showing a peak at ~4.6° confirming the presence of OCP.

XRD diffractograms of FACaP1 did not show clear peaks for CaP phases as the CaP deposits in these scaffolds were very amorphous, and XRD is effective in displaying very crystalline phases (Figure 4). FACaP1 XRD diffractogram showed peaks for mainly calcite. For FACaP2 samples, XRD diffractograms revealed peaks that could correspond to hydroxyapatite (HA) and octacalcium phosphate (OCP). However, these peaks were broad indicating the CaP mineral phase/s present were not very crystalline. Considering that the Ca/P ratio calculated by EDX is approximately 1, FACaP2 could be composed of a combination of CaP phases, for example OCP and amorphous calcium phosphate (ACP): Ca/P = 1.33 and Ca/P = 0.67–1.5 for OCP and ACP, respectively.^15^

#### Rheology

For mechanical characterisation of the material, we calculated G’ for the different scaffolds. For control FA scaffolds, G’ was 11.24 ± 2.54 kPa, for FACaP1 was 75.22 ± 55.40 kPa, and for FACaP2 was 561.33 ± 109.79 kPa. Therefore, adding a CaP mineral coating to the fibrin-based matrix strengthened the material. A plate-like morphology (FACaP2) made the scaffold stronger than a globular morphology (FACaP1).

#### Net weight and porosity

**Figure 5.**
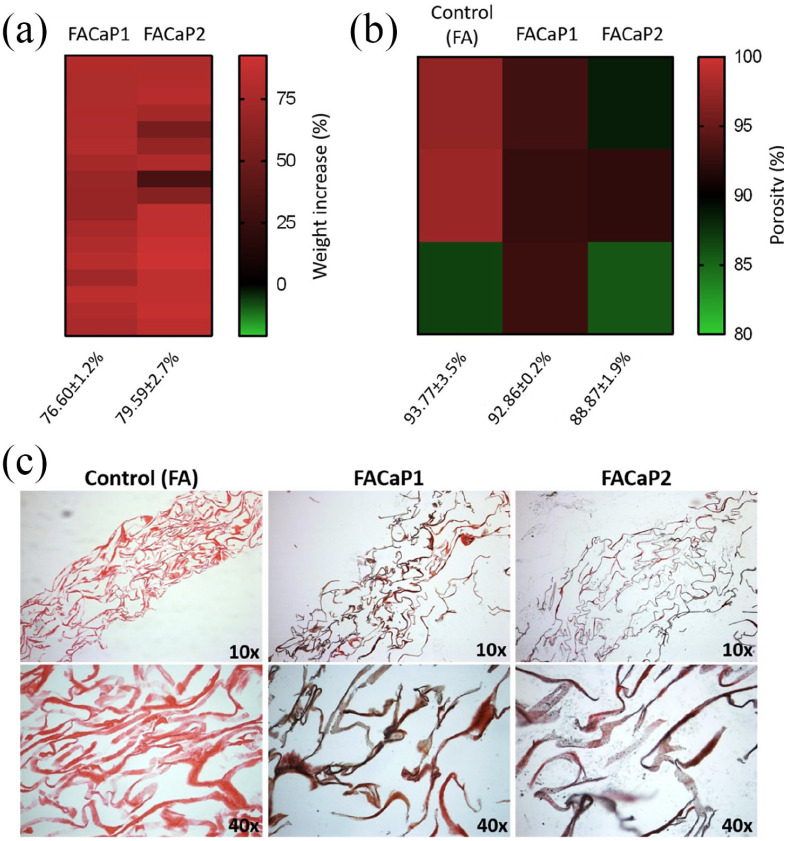
Heatmaps showing (a) the net increase in weight and (b) the overall porosity of control and FACaP scaffolds. (c) Von Kossa staining of scaffolds. Pink colour indicates Eosin staining of the FA fibres and black/brown colour indicates CaP mineral deposits.

There was a net increase in the weight of the FA scaffolds after immersion in SBF. This accounted for a net increase of 76.60 ± 1.2% for FACaP1 and 79.59 ± 2.7% for FACaP2 (mean ± SEM of multiple replicates) (Figure 5(a)).

Mercury porosimetry data (Figure 5(b)) revealed that the porosity of control FA scaffolds is 93.77 ± 3.5%, whereas CaP coated scaffolds were 92.86 ± 0.2% porous in the case of FACaP1 and, 88.87 ± 1.9 % porous in the case of FACaP2 (mean ± SEM of three replicates). This suggests that coating FA scaffolds with CaP does not significantly alter the biomaterials’ overall porosity.

#### Von Kossa staining

CaP mineral deposits were seen throughout the cross-sections of the FACaP scaffolds as indicated in Figure 5(c). CaP mineral deposits were found evenly deposited in both FACaP scaffolds and absent from the control FA scaffolds.

#### In vitro biodegradability

**Figure 6.**
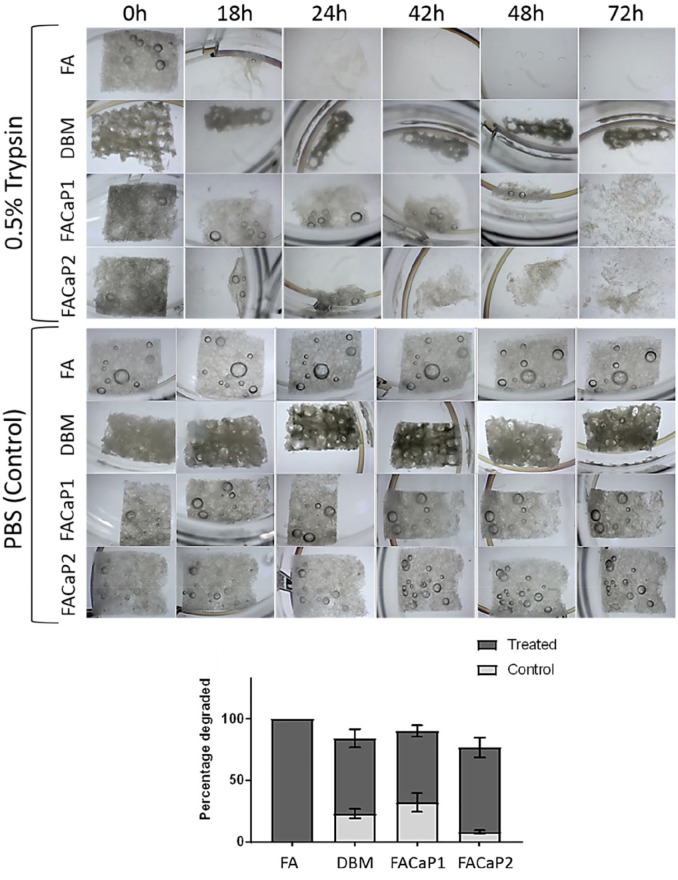
Representative images showing the degradation of scaffolds (5 × 5 mm) in trypsin-treated (top panels) versus control (bottom panels). FA scaffold was completely degraded after 72 h whereas FACaP and DBM scaffolds did not degrade completely. Superimposed histograms display the percentage of each scaffold that was degraded in treated and control conditions (mean ± SEM of *n* = 3 for each condition).

The biodegradation assay (Figure 6) results demonstrated that even after 72 h remnants of DBM, FACaP1 and FACaP2 were still seen whereas the FA scaffolds were completely degraded. These results may indicate a slower degradation time for FACaP scaffolds compared to FA ones. In control conditions, scaffolds remained intact with minimal degradation after 72 h.

### In vitro cell work

#### In vitro response of inflammatory cells

**Figure 7.**
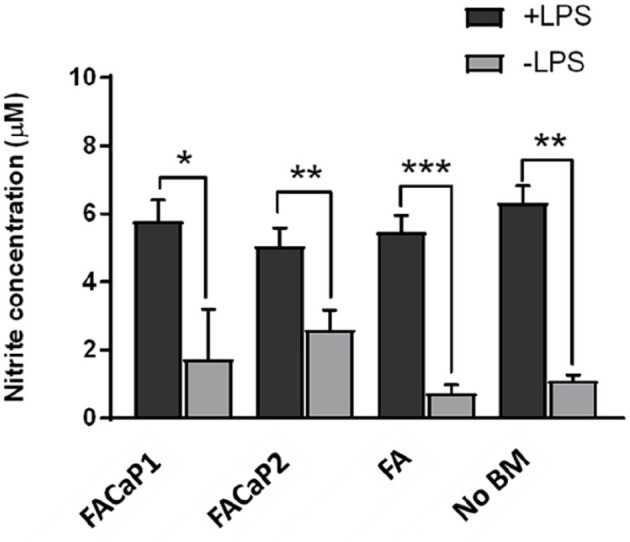
NO production by RAW264.7 cells. Under + LPS condition all conditions showed a significant increase in NO production. This increase was, however, no more than NO production by RAW264.7 cells alone (No BM: no biomaterial). No significant differences were observed between FACaP and FA scaffolds. Data are presented as mean ± SEM of *n* = 3 for each condition. *p < 0.05. **p < 0.01. ***p < 0.001.

NO production was significantly higher in all the groups treated with LPS compared to without LPS. There was no further induction of NO production by RAW264.7 cells when in contact with the biomaterial compared to no biomaterial. There was no significant difference in NO production by RAW264.7 cells when cultured on either of the scaffolds (Figure 7).

#### Cell viability and proliferation of osteoprogenitor cells

**Figure 8.**
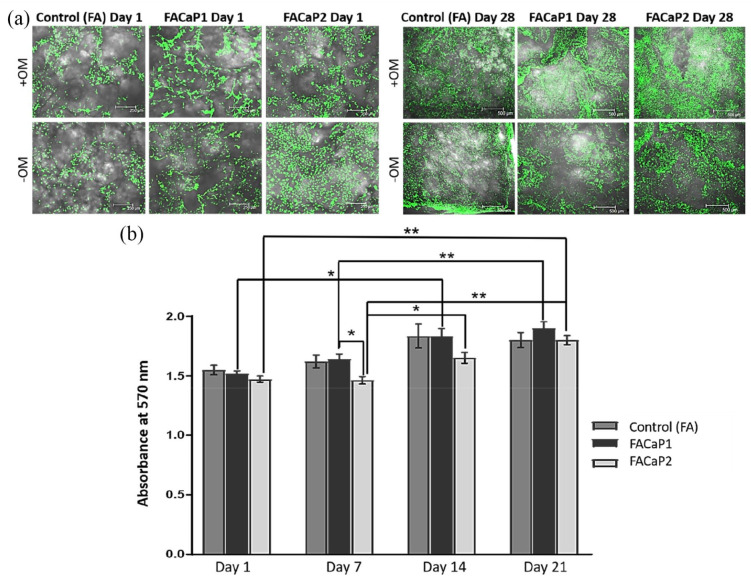
(a) Representative images of Live/Dead stained scaffolds at day 1 and day 28. Green fluorescence indicates live cells and were seen in all the scaffolds over 28 days under both +OM and −OM conditions. Note the confluent growth of cells under +OM conditions at day 28. (b) Cell proliferation in control and FACaP scaffolds (mean ± SEM of *n* = 4 for each condition). *p < 0.05. **p < 0.0001. Results show significant proliferation for cells cultured on the scaffolds over the culture period.

Results showed that cells were viable under both +OM and −OM conditions with no dead cells seen in either of the scaffolds (Figure 8(a)). From day 1 to day 21 cells proliferated as indicated by the increase in metabolic activity (Figure 8(b)) and an increase in the number of cells seen in the 3D z-stack images. A significantly higher metabolic activity was seen in FACaP1 scaffolds compared to FACaP2 scaffolds at day 7. A significant increase was also seen from day 1 to day 14 and from day 7 to day 21 for FACaP1 scaffolds. Further, a significant increase in metabolic activity was also seen in FACaP2 scaffolds from day 1 to day 21, from day 7 to day 14 and from day 7 to day 21 (Figure 8(b)). Morphologically, the cells under +OM conditions were more confluent and appeared to be growing in clusters.

#### Infiltration and migration of osteoprogenitor cells

**Figure 9.**
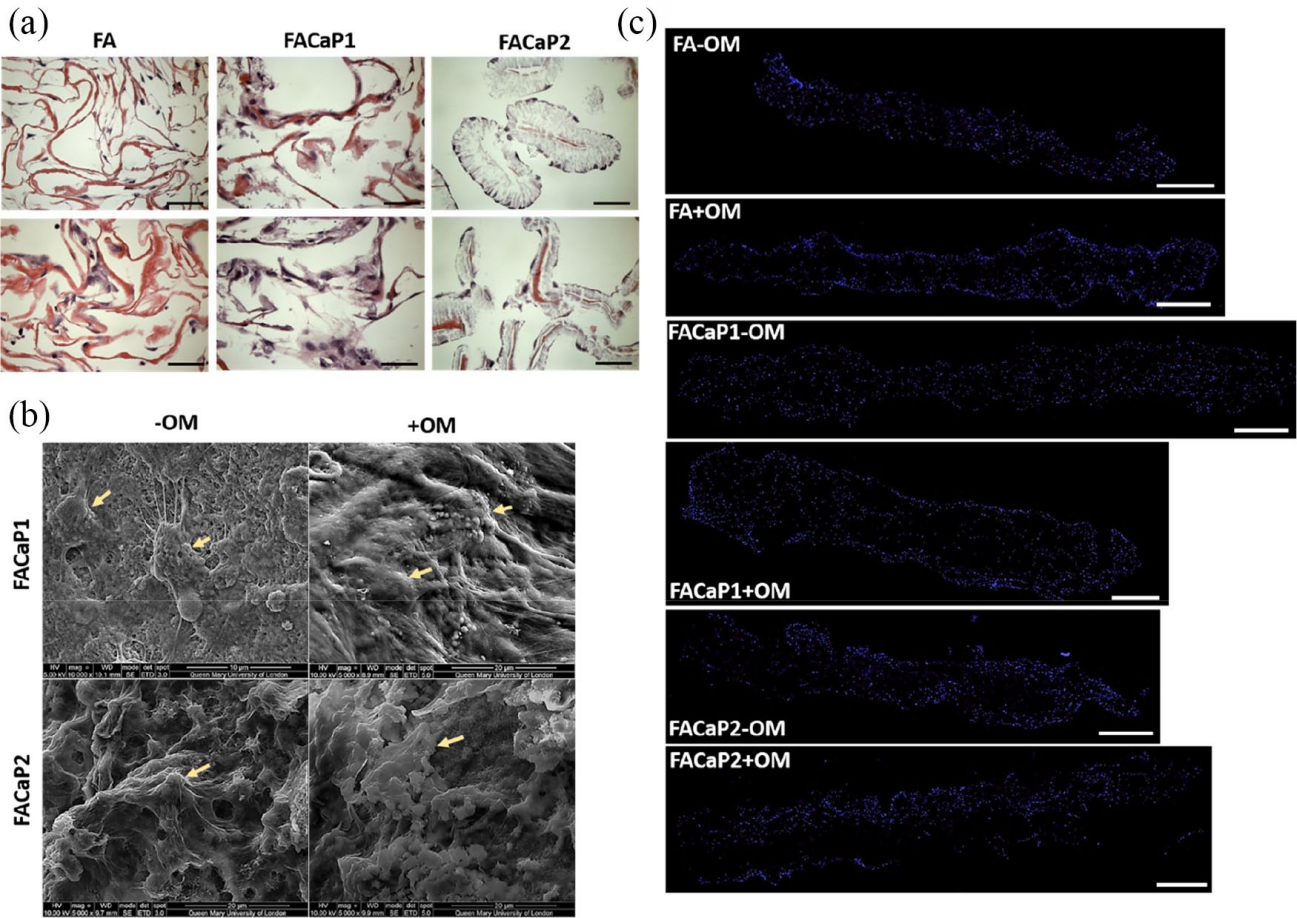
(a) Representative H&E stained images of MC3T3-E1 seeded scaffolds at day 28 of culture. Pink colour is indicative of the eosin staining of the fibres and dark purple staining is indicative of cells (bar = 50 µm). (b) Representative SEM images of MC3T3-E1 seeded FACaP scaffolds under −OM and +OM conditions at day 28 of culture. Arrows point to cells integrated within the scaffolds. (c) Representative stitched images of DAPI stained scaffolds to show cell migration throughout the scaffold after 28 days in culture. Blue fluorescence shows cell nuclei of single cells (bar = 500 µm).

MC3T3-E1 cells infiltrated the scaffold as shown in Figure 9. The cells adhered and migrated throughout the depth of the scaffolds during the 28 days in culture. Cells appeared normal phenotypically with extended filopodia and cell-processes in both −OM and +OM conditions. Cells appeared aggregated and more confluent in +OM condition. H&E and DAPI staining of MC3T3-E1 cells in FACaP scaffolds under both −OM and +OM conditions showed that the cells were homogeneously distributed throughout the scaffold, similar to the control FA scaffolds.

#### Osteogenic differentiation of osteoprogenitor cells

**Figure 10.**
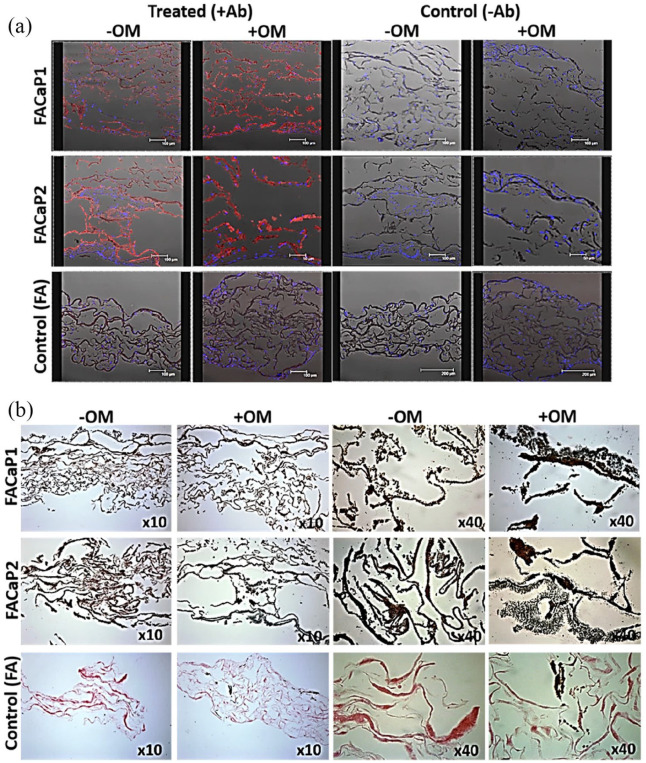
Representative images at day 28 of culture of (a) osteopontin immunostaining and (b) Von Kossa staining of cell seeded scaffolds under +OM and −OM conditions. Red fluorescence indicates osteopontin expression, blue fluorescence indicates DAPI stained cell nuclei. Osteopontin expression was clearly seen in FACaP scaffolds compared to control scaffolds and it was more abundant under +OM conditions. Black/brown colour in Von Kossa stained section indicates CaP mineral deposits. An intense staining of CaP mineral deposits was seen in FACaP scaffolds compared to FA control scaffolds.

Immunostaining of osteopontin, a protein present in the ECM of bone, showed that MC3T3-E1 cells differentiated down the osteogenic pathway in both FACaP scaffolds (Figure 10(a)). Osteogenic differentiation as indicated by red fluorescence staining was observed in FACaP scaffolds under −OM conditions; however, somewhat greater differentiation was observed in samples cultured under osteogenic conditions. Von Kossa staining revealed a marked increase in brown/black colour indicative of matrix mineralisation in FACaP scaffolds seeded with cells. FACaP2 scaffolds showed more mineralisation compared to FACaP1 scaffolds as seen by more intense brown/black colour in the former scaffolds (Figure 10(b)).

### Ex ovo CAM assay

**Figure 11.**
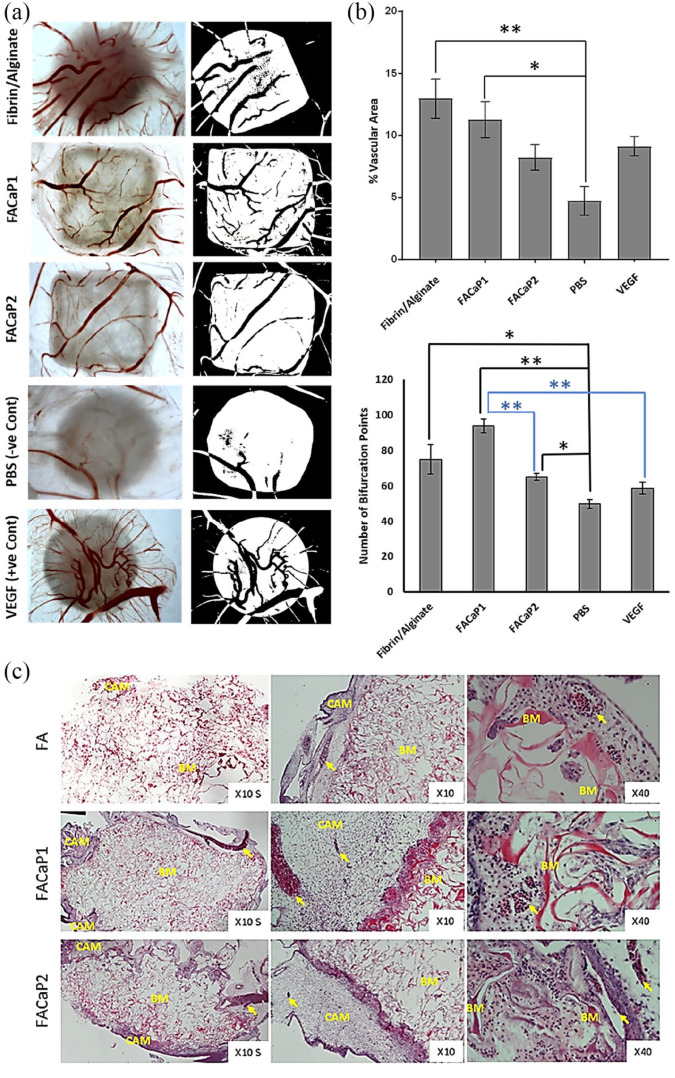
(a) Representative macroscopic (left) and binary images (right) of the different scaffolds and controls. Blood vessels are seen in red colour (left panel) and black colour over white background of the scaffold (right panel). Please note that the image for C-was already published in Kohli et al.^23^ (b) Percentage vascular area and bifurcation points of blood vessels within the scaffolds. Data are presented as mean ± SEM of *n* = 6 for each condition (except for the number of bifurcation points for FA and FACaP1 groups where *n* = 5). *p < 0.05. **p < 0.01. (c) Representative H&E stained images of FACaP and control scaffolds. Yellow arrows point at individual blood vessels. BM refers to the biomaterial and CAM refers to the surrounding CAM.

We used an ex ovo CAM assay to assess the angiogenic capacity of the FACaP and FA scaffolds. Since fibrin is pro-angiogenic, we expected a greater vascular infiltration in FA scaffolds. Results confirmed the angiogenic potential of FACaP scaffolds where blood vessels were seen to infiltrate both FACaP scaffolds from the periphery all the way to the middle (Figure 11). The percentage vascular area for FA and FACaP1 scaffolds was significantly higher than the negative control PBS sample (Figure 11(b)). No significant differences were observed between the two FACaP scaffolds. In terms of the number of bifurcation points, the three scaffolds obtained a significantly higher number than the negative control PBS. Additionally, FACaP1 had a significantly higher number of bifurcation points than FACaP2 and the positive control VEGF (Figure 11(b)). H&E staining of scaffolds (Figure 11(c)) excised from CAM membranes showed the presence of blood vessels throughout the depth of the scaffold confirming that blood vessels did not just superficially embed on the surface of the scaffolds but infiltrated them. All the scaffolds integrated well with the surrounding CAM and no foreign body giant cells were visible for any of the scaffolds.

## Discussion

In this study we developed novel fibrin-based composites intended as scaffolds in bone tissue engineering applications. A novel aspect of our proposed scaffolds is that they are made of fibrin and alginate. The base FA material was developed in our laboratory for the treatment of full-thickness skin injuries. This biomaterial has been extensively studied by our group and we have repeatedly shown its excellent cell adhesion and cell proliferative properties.^11,12^ Because of the advantages associated with the FA matrix, we intended to explore if it would be suitable for bone regeneration. For this purpose, we used the SBF immersion method to biomimetically coat the FA scaffold with CaP. Since fibrin is a pro-angiogenic protein and alginate contains many carboxylic acid groups, which aid heterogenous mineral nucleation, we hypothesised that together they could make a candidate biomaterial for bone applications. Coating biomaterials using SBF has been used for a variety of materials including metals,^24,25^ glasses^26^ and polymers^27–30^; however, to the best of our knowledge, a fibrin-based biomaterial has never been used.

The two prototypes, FACaP1 and FACaP2 both contain a mineral coating representing a mixture of phases. CaP coatings have been widely studied for bone regeneration applications due to their potential for osteoconduction and in some cases osteoinduction.^15,31–33^ In FACaP1, the globular morphology of CaP is indicative of an amorphous calcium phosphate (ACP) phase containing Mg^2+^ ions, whereas for FACaP 2, the plate-like morphology of CaP may be indicative of OCP phase as well as HA. Taking into account the ratio of Ca/P for both prototypes, it can be conferred that both prototypes represent a mixture of CaP phases. In fact, both prototypes showed a lack of crystallinity and therefore, represent precursor CaP phases for subsequent bone mineralisation. Previous studies have shown that ACP can act as a transient phase which can easily be transformed to apatite via multiple intermediate stages.^15,34^ It has been shown before that the phase transformation in aqueous solutions occurs through dissolution of the parent phase followed by nucleation and growth of the new phase.^35,36^ In our study, FACaP1 was immersed in 5× concentrated SBF solution for 24 h, which led to the formation of a pre-nucleation complex. Further the EDX spectra revealed the presence of Mg, which inhibits crystal formation. It may be speculated that the pre-nucleation complex in FACaP1, when immersed in SBF-2 solution, which lacks Mg^2+^ ions and HCO_3_^–^ ions, results in the formation of an OCP phase. Previous studies are consistent with our findings that HA formation in vitro and in vivo proceeds through a phase transformation from ACP to DCPD to OCP and finally to HA.^37^ For instance, Chahal et al.^17^ recently reported a composite of hydrogel poly(ethylene glycol) with ACP, where ACP transformed to HA via OCP after flushing with 100 mM Tris-HCl buffer pH 7.4. In our study, the phase transformation occurred through immersions in two different 5× concentrated SBF solutions as previously described.^32,38,39^

The deposition of CaP in both FACaP1 and FACaP2 was observed throughout the depth of the scaffolds. This may be due to the high porosity of the FA matrix, which was not compromised even with CaP coating as shown by MIP results. The high interconnectivity of the pores has been shown to be an essential requirement for appropriate cell adhesion and proliferation.^40^ However, with increasing porosity, the mechanical strength of the biomaterial is often reduced. Both FACaP1 and FACaP2 scaffolds showed a higher viscoelastic modulus (G’) compared to the FA scaffold alone, while maintaining their high porosity. Further there was a net increase of over 70% in the dry weight of the scaffolds due to CaP coating. Together with Von Kossa staining, it can be conferred that an even coating of CaP was achieved on the scaffolds that allowed for increased stiffness without compromising the porosity of the scaffolds. For bone regeneration, a high overall porosity in conjunction with a gradient pore-size range is a pre-requisite to allow for vascular infiltration and cell-adhesion and proliferation.^40–42^

It has been shown that proteases play an important role during bone healing and bone remodelling.^43,44^ When implanted in vivo, FACaP scaffolds would meet the proteases present in the bone healing environment. Therefore, the serine protease trypsin was used in this study to mimic the in vivo environment. We used a high concentration of trypsin in order to accelerate the degradation process. The degradation rate of FACaP scaffolds was closer to DBM, indicating a slower degradation time due to the addition of CaP in the FA matrix. DBM is currently used clinically as a bone graft substitute; however, its use is limited by difficulty in manufacturing the scaffold, not being cost-effective and lacking angiogenic potential.^45^

Since bone remodelling is a dynamic process involving many different cell types including inflammatory cells,^22^ we used RAW264.7 cells to assess NO production as a model of inflammation. While it is challenging to simulate the complexity of host immune response elicited by a biomaterial in an in vitro model, the assay used in this study gives an indication of the initial immune response by macrophages and allows the comparison of this response between biomaterials. We did not see an enhanced production of NO by RAW264.7 cells in either of our scaffolds. This may be due to the composition of the scaffold, that is, fibrin which has been shown to have a protective effect on macrophages, preventing a severe inflammatory response.^46^

MC3T3-E1 cells on both prototypes, adhered, proliferated and migrated throughout the scaffold during the culture period of 28 days. Therefore, by coating the FA matrix with CaP, the scaffold still maintains its cell adhesion, migration and proliferation properties exhibited by FA in our previous work.^11–14^ Our results showed that cells directly attach to the CaP coating. The fact that cells were seen throughout the depth of the scaffolds shows that the porosity is optimal for cell infiltration. In a clinical scenario, this would mean that post-implantation, host cells would be easily able to penetrate and migrate through the scaffold and allow healing to take place. Further, the cells may be remodelling the CaP coated FA matrix with increased expression of osteopontin in FACaP scaffolds compared to the control scaffolds: even in the absence of osteogenic supplements, osteopontin expression was evident in FACaP scaffolds. It has previously been shown that CaP coatings, depending on their phase, may either suppress or enhance osteoblast cell proliferation and differentiation.^47–50^ Chou et al.,^49^ showed that large plate-like structures of CaP coating significantly induced a higher expression of osteogenic markers compared to precursor phases. Another study showed that biomimetic carbonated apatite phase is most favourable for MC3T3-E1 cell proliferation and differentiation.^51^ These studies imply that the difference in osteoblast cell behaviour in vitro depends largely on the physicochemical properties of the CaP coatings as well as the surface topography and roughness of the substrate for cell-attachment.^52^ Both FACaP1 and FACaP2 allowed cell attachment, proliferation and differentiation. However, at an early time point, (day 7) FACaP1 showed a significantly higher cell proliferation compared to FACaP2. OCP, which promotes osteoblastic cell differentiation in vitro and bone regeneration in vivo,^18^ has been previously shown to induce a higher cell proliferation only after day 7, similar to our results, where FACaP2 showed a significant increase in the metabolic activity of MC3T3-E1 cells from day 7 in culture.^51^ The trend for MC3T3-E1 cell proliferation after day 7 was lower on FACaP2 scaffold compared to FACaP1 scaffolds. It may be speculated that the presence of OCP and/or HA phase in FACaP2, is inducing a switch from a proliferative state to a differentiated state. This is consistent with previous findings where a higher expression of osteogenic differentiation markers is seen on more matured CaP phases compared to precursor phases.^47,48,51^

A recent study showed that fibrin along with biphasic calcium phosphate acts as an excellent composite material for bone regeneration compared to biphasic calcium phosphate alone, both in vitro and in vivo.^53^ Our proposed biomaterial with fibrin as the base material might show beneficial results for bone regeneration in vivo, too. Our ex ovo CAM assay showed that FACaP prototypes had enhanced capacity for angiogenesis. The FDA approved the use of CAM assays as a suitable model for testing biomaterials pre-clinically; however, only a handful of studies have used this method to examine initial tissue response to biomaterials.^54–57^ In our group, we use CAM assays to assess the angiogenic capacity and biocompatibility of our biomaterials.^23^ Both our prototypes are biocompatible as indicated by the absence of foreign body giant cells, as their presence is indicative of a foreign body reaction leading to implant failure.^58–60^ Further, both prototypes grafted well within the CAM mesenchyme, suggesting that the biomaterials were well-tolerated by the host tissue. In a CAM assay, blood vessels penetrate from the edges of the scaffold towards its centre,^23^ and it is representative of the vascularisation that would occur in vivo upon implantation of the scaffold. Quantification of the percentage of vascular area is a measurement of the extent of blood vessel infiltration.^23,54–58^ The blood vessel infiltration in our scaffolds was dependent not just on the composition of our biomaterial, but also on the porosity, as the filter disc soaked in VEGF did not show a higher vascular density compared to fibrin-based scaffolds.^23^ This is because of the high porosity of the scaffolds compared to the extremely low porosity of the filter disc. Adequate porosity and pore-size of the biomaterial are critical for bone formation both in vitro and in vivo, mainly to allow vascular infiltration and osteogenic differentiation.^40,61,62^ Both FACaP1 and FACaP2 provide sufficient porosity to allow cell and vascular infiltration. Additionally, bifurcation points are indicative of the vessel sprouting phase of the angiogenesis process, where pre-existing blood supply leads to vascular sprouting that then develops into mature blood vessels.^23^ Therefore, the scaffolds would encourage vessel sprouting culminating into the formation of mature blood vessels. Particularly FACaP1 showed increased vessel sprouting potential, which could be due to the morphology of the CaP deposited on this material, which is easier to dissolve than the CaP morphology observed in FACaP2 as observed in our biodegradation assay. This faster dissolution process would make calcium ions more readily available, which are known to promote angiogenesis. It has been described in the literature that cellular phenotypic changes that take place during angiogenesis need calcium ion stimulation of gradient shifts.^63^ In summary, it has been shown that coupling of angiogenesis and osteogenesis is a key requirement for successful bone regeneration.^64^ Therefore, combining fibrin with CaP has enhanced potential for bone regeneration.^3^

## Conclusion

We conclude that the proposed novel pro-angiogenic and osteogenic FACaP presented in this study appears very promising in their potential as scaffolds for bone tissue engineering as an alternative to bone grafts. FACaP can be easily manufactured using simple techniques and is very cost-effective. The work presented here gave useful insights into the potential of this biomaterial and merits further research in a pre-clinical model of bone defects prior to its clinical translation.
